# Paraoxonase-1 and Other HDL Accessory Proteins in Neurological Diseases

**DOI:** 10.3390/antiox10030454

**Published:** 2021-03-15

**Authors:** Judit Marsillach, Carlo Cervellati

**Affiliations:** 1 Department of Environmental & Occupational Health Sciences, University of Washington, Seattle, WA 98105, USA; 2 Department of Morphology, Surgery and Experimental Medicine, University of Ferrara, 44121 Ferrara, Italy

The burden of neurological diseases continues to increase as they still are the leading cause of disability and the second-leading cause of death worldwide [1]. In the United States, Alzheimer’s disease (AD) and other dementias such as vascular dementia (VD), and multiple sclerosis are among the five leading causes of death from neurological diseases [2]. The central nervous system (CNS) is highly metabolic, resulting in high consumption of oxygen that leads to production of reactive oxygen species (ROS). ROS induce progressive damage to DNA, lipids, carbohydrates, and proteins, resulting in losses of physiological functions [3,4]. Oxidative stress, an imbalance between the production of ROS and the ability to detoxify ROS and repair its damage, has been implicated in the pathogenesis of several neurological disorders. The antioxidant defense system can prevent oxidative damage, but its efficiency progressively declines with aging.

High-density lipoproteins (HDL) are a heterogeneous group of lipoproteins composed of lipids and proteins that possess a wide range of functions, including antioxidant and anti-inflammatory functions, in addition to cholesterol transport. HDLs are found in the systemic circulation, although the lipoproteins found in the CNS are considered HDL-like as they also contain apolipoprotein (Apo) A-I, enzymes, transporters, and receptors similar to plasma HDLs (with the difference being that they are enriched in Apo E instead of ApoA-I) [5]. Mounting evidence indicates that the benefits of HDLs expand beyond the cardiovascular system, modulating cognitive function in aging and age-related neurological disorders [6,7,8,9,10,11]. The lipid and protein composition of HDLs influences their function. In particular, changes in the protein constituents that negatively affect HDL functionality have been repeatedly found to increase the risk of CNS disorders [12,13,14,15]. This Special Issue focuses on the HDL proteome and its role in neurological diseases.

The article by Marsillach et al. [10] provides an exhaustive review of the main protein determinants of HDL’s biological function, the antioxidant enzyme paraoxonase-1 (PON1) and selected apolipoproteins, including ApoA-I, -E, and -J, and their role in AD. These Apo, with the exception of ApoA-I, are also synthesized in the brain. It is hypothesized that ApoA-I and PON1 enter the CNS via the blood–brain barrier as discoidal HDLs, in a still-under-debate mechanism [16,17,18]. Additionally, the authors also highlight the importance of HDL functionality based on its protein cargo over the classic HDL hypothesis that increasing HDL cholesterol decreases the risk of cardiovascular disease. This should be kept in mind for future studies focusing on the CNS HDL-like lipoproteins, as the HDL protein cargo may prevent the aberrant changes in the brain that characterize AD pathogenesis and other neurological diseases.

Of particular interest is PON1, a potent antioxidant, anti-inflammatory, and anti-apoptotic enzyme found in circulation in HDLs. Studies of PON1 in neurological diseases are limited [19], in comparison to other oxidative stress-related diseases such as cardiovascular disease, mostly due to the assumption that its antioxidant activity is limited to the circulating lipoproteins. However, as highlighted by Reichert et al. [20], increased oxidative stress and decreased PON1 activity has been strongly associated with the pathophysiology of several neurological diseases, including multiple sclerosis, amyotrophic lateral sclerosis, AD, and Parkinson’s disease (PD). The authors provide a detailed review of the reports on PON1 in these diseases and conclude that robust studies at the PON1 polymorphic level and at the PON1 cellular level are still missing and are necessary to understand the physiological function of PON1 in the neurodegenerative process. It should be noted that the majority of studies on the effects of PON1 polymorphisms on disease have disregarded the most important factor that determines susceptibility and risk to disease, which is PON1 levels and PON1 functionality, leading to contradictory results [10]. Piras et al. studied serum PON1 arylesterase activity in neurodevelopmental disorders, more specifically in autism spectrum disorder (ASD) and in attention deficit/hyperactivity disorder (ADHD) [21]. The authors reported significantly decreased serum PON1 arylesterase activity only in children and adolescent ADHD patients, compared with controls, independently of any of the PON1 gene variants genotyped (SNPs rs705379 and rs662).

A few reports have indicated that PON1 is present in cerebrospinal fluid (CSF) [19,22,23,24] and in certain areas of the mouse brain [25,26], providing more evidence of a potential role of PON1 in neurological diseases. Two of these reports are included in this Special Issue. The study by Romani et al. is the first one to compare activity levels of PON1 in serum and CSF of a large cohort of patients with vascular dementia (VaD) and late-onset AD (LOAD), compared to control subjects [24]. They found decreased PON1 arylesterase activity in plasma and CSF of VaD and LOAD patients, compared with controls. Additionally, the authors propose the use of PON1 arylesterase activity/ApoA-I ratio as a potential biomarker for monitoring AD progression, based on the reported results with this ratio predicting total tau, a marker of neurodegeneration, in AD patients. On the other hand, the study by Salazar et al. is the first report showing PON1 (and PON3) protein expression in glia cells surround amyloid-β plaques in one of the most widely used mouse models of AD disease (Swedish Tg2576) [26]. Although the results from this study are preliminary and warrant further examination to ascertain which brain cell types contain PON1 and PON3, they reinforce the hypothesis that HDLs act as delivery carriers of PON1 and PON3 from the liver to areas of high levels of oxidative stress and inflammation, suggesting that PON1 and PON3 may cross the blood–brain barrier (as there is no known PON1 and PON3 gene expression in brain) [25,27,28,29,30], and provide further evidence of a potential role played by the PON family members in AD and other neurodegenerative diseases.

We would like to acknowledge all the authors that have contributed to this Special Issue with reviews and original research. From the collection of publications, it is clear that further research into the role and mechanism of protection of HDLs and their protein cargo in neurological diseases has the potential to lead to novel biomarkers of disease and progression, as well as to novel clinical interventions.
